# Birdsong Denoising Using Wavelets

**DOI:** 10.1371/journal.pone.0146790

**Published:** 2016-01-26

**Authors:** Nirosha Priyadarshani, Stephen Marsland, Isabel Castro, Amal Punchihewa

**Affiliations:** 1 School of Engineering and Advanced Technology, Massey University, Palmerston North, New Zealand; 2 Institute of Agriculture & Environment, Massey University, Palmerston North, New Zealand; 3 Asia-Pacific Broadcasting Union, Kuala Lumpur, Malaysia; The Australian National University, AUSTRALIA

## Abstract

Automatic recording of birdsong is becoming the preferred way to monitor and quantify bird populations worldwide. Programmable recorders allow recordings to be obtained at all times of day and year for extended periods of time. Consequently, there is a critical need for robust automated birdsong recognition. One prominent obstacle to achieving this is low signal to noise ratio in unattended recordings. Field recordings are often very noisy: birdsong is only one component in a recording, which also includes noise from the environment (such as wind and rain), other animals (including insects), and human-related activities, as well as noise from the recorder itself. We describe a method of denoising using a combination of the wavelet packet decomposition and band-pass or low-pass filtering, and present experiments that demonstrate an order of magnitude improvement in noise reduction over natural noisy bird recordings.

## Introduction

More than 13% (1,373) of bird species are vulnerable or in danger of extinction from causes such as deforestation, introduction of alien species, and global climate change (International Union for the Conservation of Nature Red Data List, 2014). In order to conserve bird populations, wildlife managers require accurate information about species presence and population estimates derived from monitoring programmes. Although birds are hard to spot visually even when the observers are in the correct place, they are more vocal than other terrestrial vertebrates and therefore birdsong is usually the most direct way for humans to detect them. With the development of acoustic recorders that can be left in the field for extensive periods of time capturing all songs, including rare ones, traditional call count surveys are being replaced by the collection of terabytes of data, which can be collected relatively cheaply and easily with limited human involvement.

The permanent storage of this acoustic data brings the advantage of being able to listen to the songs and to view their spectrograms again and again, improving the accuracy of both species recognition and call counting. However, this work is still largely manual, requiring spectrogram reading and listening, which makes it a costly approach that requires well-trained individuals; it reportedly takes an expert approximately one hour to scan the spectrogram of ten hours of recording [1] (depending on the quality of the recordings, species being monitored, and call rate), which is a daunting task, especially when many recordings are often collected simultaneously [2]. Consequently, sampling (analysis is done on limited time periods within a subset of recordings) is favoured in many surveys, but it introduces bias and incompleteness, hence the desire to automate the recognition of bird species from their songs.

Compared to human speech recognition, one of the principle challenges of birdsong recognition—which obviously occurs in natural environments—is noisy recordings. The recorder picks up all of the noise that is in the environment, not just the birdsong, and the birds are rarely very close to the microphone. This leads to a low signal-to-noise ratio, making it hard to even detect the birdsong, let alone recognise it, whether the recognition is done by human or computer. In this paper we consider the problem of denoising birdsong from a signal processing point of view. We discuss what makes up the various types of sound that birds emit, and then the sources of noise that can be present. We then consider the signal processing methods that are available, and compare two methods: a traditional approach based on band-pass/low-pass filtering, and our own, which uses the wavelet packet decomposition in concert with band-pass or low-pass filtering. Using songs and calls from different bird species (that cover a range of vocalisations and frequencies), we demonstrate that we can significantly improve the quality of recorded birdsong, both individually segmented, and over relatively long periods.

## Bird Vocalisation, Categorisation and Spectrogram Patterns

Bird vocalisations play a major role in species-specific communication, including mate attraction, parent-offspring interaction, cohesion among flocks, and territorial defence [3]. Experiments have shown that birds are capable of recognising conspecifics, individuals, and other species using songs alone [4]. Each bird species has their own song repertoire, which can vary from monotonous repetition to innovating new, complex songs (for example, the superb lyrebird (*Menura novaehollandiae*) and brown thrasher (*Toxostoma rufum*) [5]).

**Fig 1:**
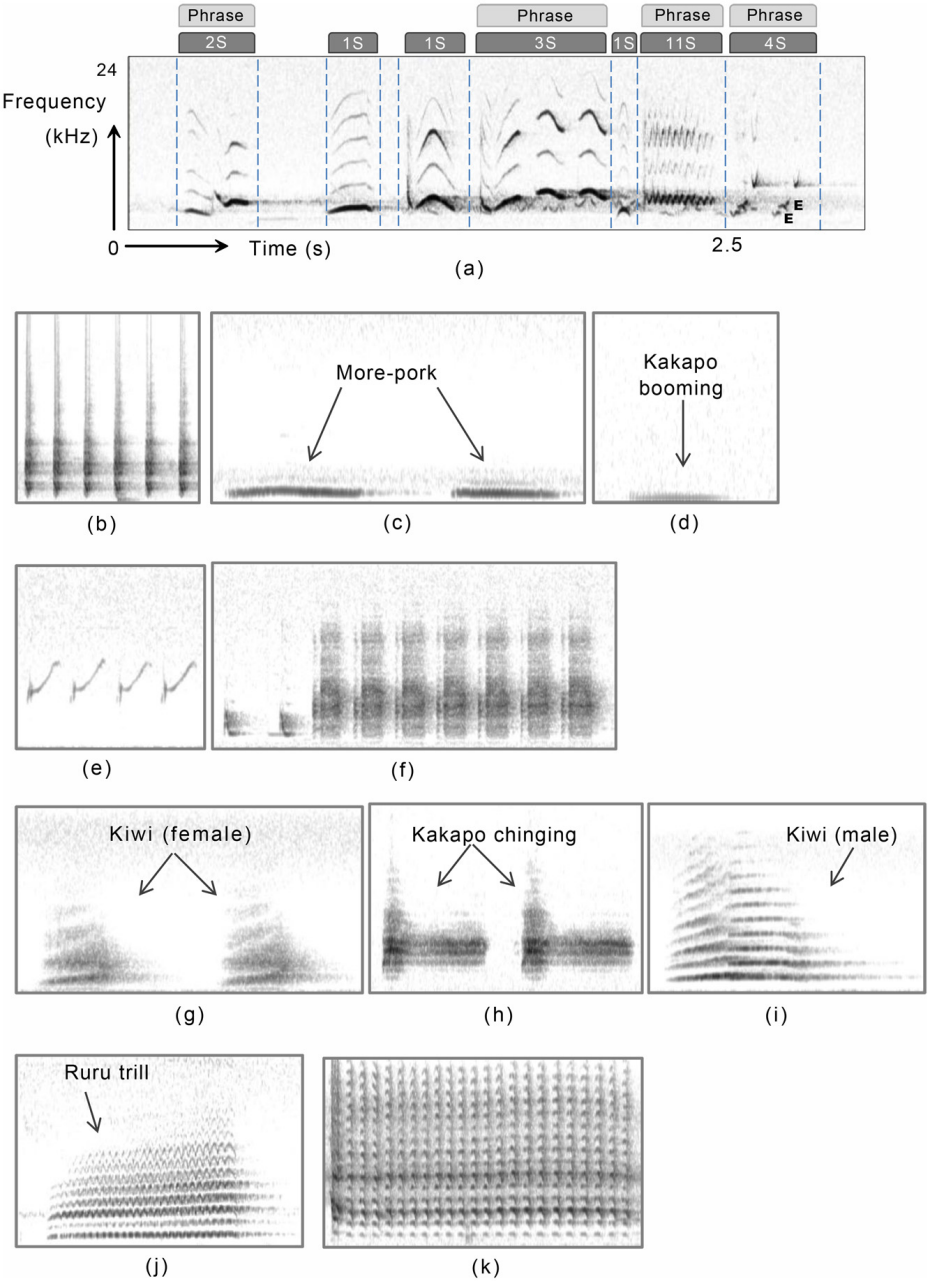
Spectrogram representations of various bird species showing some of the typical appearances of sounds. (a) A fox sparrow (*Passerella iliaca*) song illustrating its syllables, phrases, and elements (S = syllable and E = element). (b)-(e) show representations of lines: (b) tui (*Prosthemadera novaeseelandiae*); (c) the *more-pork* sound of ruru (*Ninox novaeseelandiae*); (d) kakapo (*Strigops habroptilus*) *booming*; (e) brewer’s sparrow (*Spizella breweri*). (f)-(h) demonstrate blocks: (f) (long billed) marsh wren (*Cistothorus palustris*); (g) female North Island brown kiwi (*Apteryx mantelli*) call; (h) kakapo *chinging*. (i)-(j) show stacked harmonics: (i) male North Island brown kiwi whistles; (j) ruru *trill*. (k) oscillations: North Island saddleback (*Philesturnus rufusater*).

Vocalisations can be categorized into calls and songs, where calls are composed of fairly simple sounds produced by both sexes, while songs are long and complex and produced more commonly by male songbirds (order *passeriformes*). The main difference between calls and songs is arguably their function: songs are generally viewed as having a role in reproduction, while calls have an ever increasing number of functions from territoriality, to individual identification, to communicating complex messages such as type and size of predator presence [6–8]. Songs and calls can be further divided into phrases, syllables, and elements [9], as shown in Fig 1(a). The fundamental unit of sound is the element, with syllables being comprised of one or more elements that can be separated from the other content of the vocalisation. A series of syllables that are organised into some pattern is referred as a phrase.

There are a number of studies that define the components of bird vocalisations based on the patterns they generate in a spectrogram [10, 11]. The key acoustic components defined by [10] are lines (at any angle), warbles, blocks, oscillations and stacked harmonics (examples are given Fig 1(b)–1(k)).

### Spectrogram Analysis

The spectrogram representations of birdsong shown in Fig 1 are based on the frequency representation of a discrete recording of the continuous birdsong. Digital recording of birdsong is based on equally-spaced time sampling of the analogue birdsong. This primary form of acoustic data is referred to as the oscillogram or simply the waveform. The oscillogram is two dimensional: the horizontal axis represents time and the vertical axis represents amplitude. It turns out that signal analysis is generally more effective in the frequency domain than in the time domain, as is evidenced by the fact that ornithologists prefer the spectrogram representation to the oscillogram one. The frequency representation provides information about the frequency components that comprise the signal, but not about when those frequencies occur. Converting the waveform into the frequency domain is performed by the Fourier transform, which represents the signal as a weighted combination of sine and cosine waves at different frequencies. The Fourier transform is invertible, meaning that processing can be performed in the frequency domain and then transformed back into the time domain, for example to enable the sound to be played. Birdsong is transferred into the frequency domain by applying the Discrete Fourier Transform (DFT), and in practice the Fast Fourier Transform (FFT), which is a computationally efficient algorithm for the DFT, is used.

**Fig 2:**
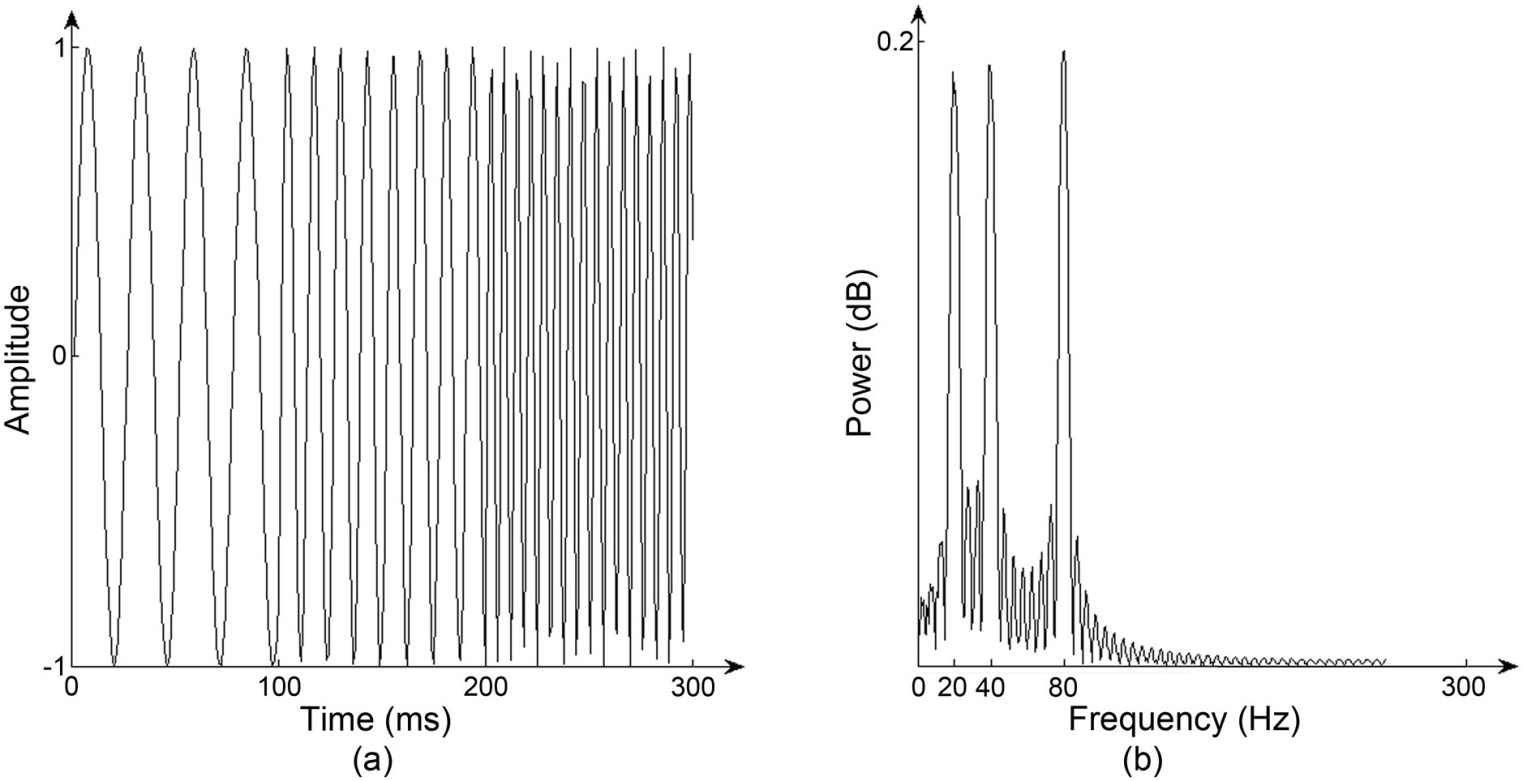
Non-stationarity. (a) A non-stationary signal containing 20 Hz, 40 Hz and 80 Hz frequencies and (b) its power spectrum computed using the Discrete Fourier Transform.

Fig 2(a) shows a non-stationary signal. During the first 100 ms the frequency of the signal is 20 Hz, during the second 100 ms the frequency doubles and again during the last 100ms. The right of the figure shows the *power spectrum*, which plots the energy per time unit (power) against the frequency components, and which clearly shows the basic frequencies of the original signal. Thus the power spectrum is a good representation of sound, summarising its periodic structure. However, it is suitable only for stationary signals while most signals in the real world are transient (non-stationary). The reason for this is that the signal is assumed to be infinite in time, and choosing a short time window has the effect of causing aliasing, where signal from outside the chosen range affects the appearance inside the range.

Segmenting the entire signal into fixed size small time windows and then calculating frequency components from these windows is a common practice based on the assumption that the signal is stationary over a short duration. Careful use of windows that decay to zero at the edges of their range and overlapping the windows enables Short Time Fourier Transformation (STFT) to be used, and this is the basis of the spectrogram. First, the power spectrum of each window is calculated, and then rotated 90° clockwise, and the amplitude is replaced by a greyscale. The complete spectrogram is generated by stacking all those images of subsequent windows appropriately. Provided that the time windows are short enough that the frequency components are stable in the time window this provides a faithful representation of the frequency components of the data against time, but it comes at a cost, since estimating frequencies accurately requires time: frequency resolution can only be achieved at the cost of time resolution and vice versa. The result of this is that larger windows are required for low frequencies, but STFT cannot deal with these subtleties. This led us to consider wavelets as a representation of birdsong, as we shall discuss after we consider the types of noise that are present in birdsong recordings.

## Bird Recording and Noise

Until recently, manual (attended) recording was the method of choice for recording birdsong. This generally enables the capture of good quality close-range songs provided the recordist has the skills not only to tune and handle the recorder, but also a good knowledge of the bird being recorded and how to approach it closely. The advent of waterproof programmable recorders with good battery life and high recording capacity has enabled a new form of birdsong recording, enabling ecologists to collect every sound in the forest (or other area of interest) without disturbing the birds or requiring groups of experts to perform call counting in the field. However, recordings made in natural environments are highly susceptible to a variety of noises. During attended recordings, some noise can be controlled by careful screening, but in automatic recording this is impossible.

### Types of Noise

**Fig 3:**
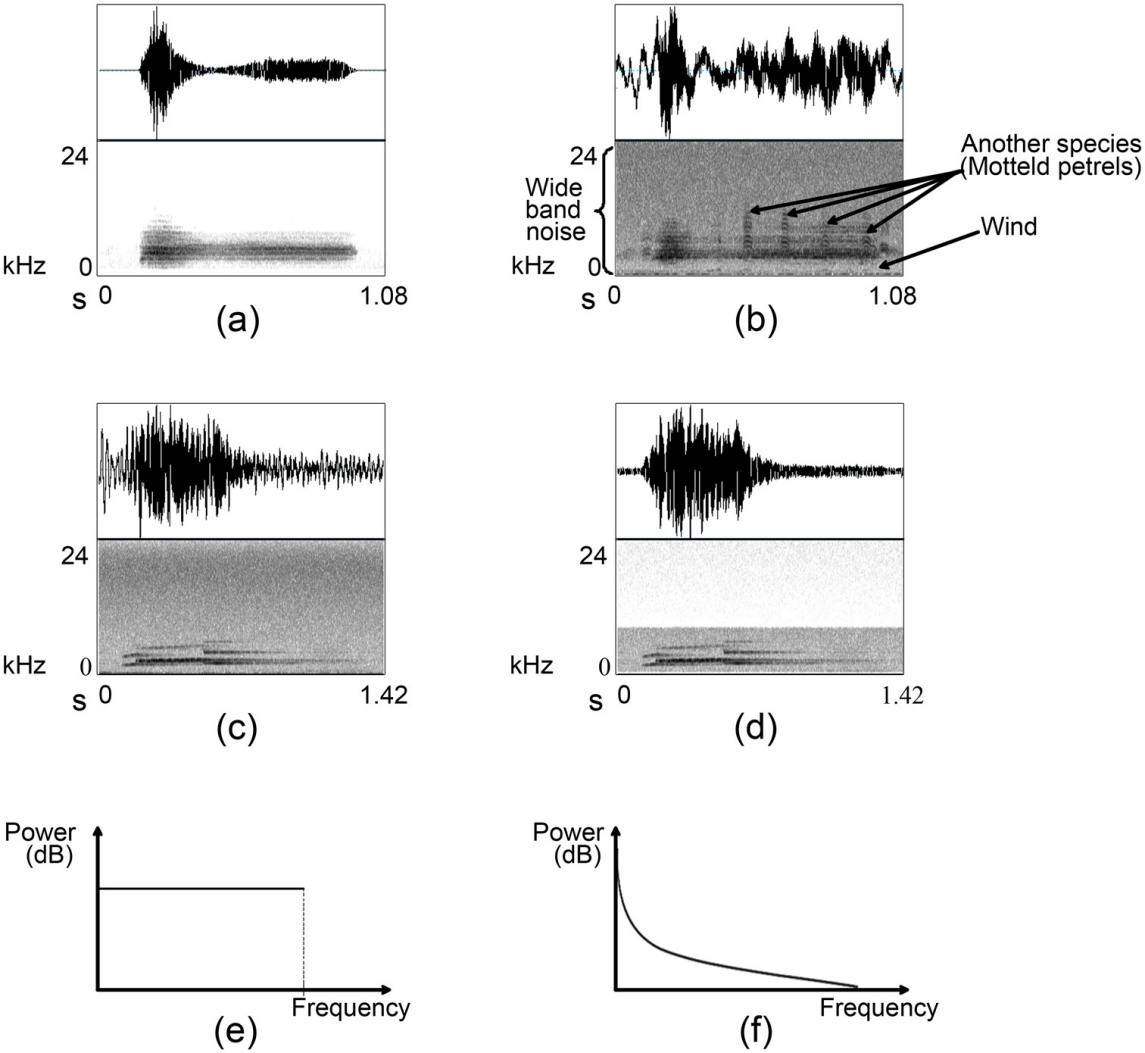
Examples of bird calls with various degrees of noise, the effect of band-pass filtering and power spectrum of white and pink noise. The top row of each sound figure displays the oscillogram and the second row the spectrogram. (a) A less noisy example of kakapo *chinging* with limited noise and (b) a noisy example of kakapo *chinging*. (c) An original male kiwi whistle and (d) its noise filtered (band-pass) signal. Noise is visible as a grey background in the spectrogram surrounding the sound depiction and most of the high-frequency variation in the oscillogram. Power spectrum of (e) white noise and (f) pink noise.

The sounds that can be heard can be categorised into three broad types: biophony, geophony, and anthrophony [12]. Biophony refers to any sound produced by biological agents: in the forest major biophonies are birds, insects, frogs/toads, and mammals. Because we are only interested in acoustic activity of birds, all other biological sounds are categorised as noise; with recordings targeted at particular bird species, even other birdsong is regarded as noise. Geophony refers to all non-biological, natural sounds in the environment such as wind and its effect on trees, rain, thunder, and running water. Field recordings are always blended with these geophonies. Anthrophony refers to all sound generated from human-made machines such as aircraft, vehicles, wind turbines, and the recording device itself: there is always some microphone and recorder hum. Collectively, these noises contaminate all acoustic data to a greater or lesser extent, see Fig 3(a) and 3(b). The problems of noise are both that it can mask the signal of the bird call, and also transform it so that it looks different, making it hard to identify. While there is some research on features that are invariant to noise, meaning that they look the same even in noisy data, they are not general, and we will not consider them further here.

We differentiate between denoising of a signal, which is principally the removal/filtering of consistent noise, from source separation, which is identifying that there are several birds calling simultaneously and separating the signals into individual birds. We do not consider the second further in this paper; [13] provides a survey of approaches to the problem, but notes that very few of the methods have been shown to work for real-world signals.

There is a theory of noise in digital signal processing (see, for example [14]), which characterises the noise according to its properties into:

**White noise** has equal energy at all frequencies, meaning that the power spectrum is flat. In practice, noise is only white over a limited range of frequencies (Fig 3(e)). While not all white noise is Gaussian, natural white noise can often be modelled as such.
**Coloured noise** shows a non-uniform power spectrum, with the energy generally decreasing in proportion to the frequency *f*. Common types of coloured noise include pink (power $\propto \frac{1}{f}$) and brown (power $\propto \frac{1}{f^{2}}$).
**Impulsive noise** refers to sudden click like sounds that last for a very short period of time (milliseconds), such as switching noise. An ideal impulse generates a horizontal line in the power spectrum because these sharp pulses contain all frequencies equally.
**Narrow-band noise** such as microphone hum shows a small range of frequencies.
**Transient noise** is a burst of noise that occurs for some time, and then disappears.

An important property of any sound is whether or not it is stationary i.e., its properties do not change substantially over time. Most noise in natural recordings is at least quasi-stationary, being geophonic in nature. However, birdsong is not stationary (i.e., it is transient) since it is generally short-lived and varies quickly. This difference between the properties of the noise and signal enables noise reduction techniques to be applied.

### Noise Filtering

Noise filtering is the most common approach to dealing with noisy recordings. Traditional signal processing, based on electronics, uses two basic filters, low-pass and high-pass, which allow frequencies respectively below and above a pre-defined cut-off frequency to pass through, and attenuate the rest. Combining a low-pass filter and a high-pass filter gives a band-pass filter. If the noise occupies high frequencies while the bird of interest sings low frequency songs then this would be sufficient to eliminate noise, but since the spectra of the noise and the signal overlap, this is not the case.

Fig 3(c) and 3(d) illustrates the effect of band-pass filtering on a single instance of a male North Island brown kiwi (*Apteryx mantelli*) whistle. The spectrogram shows that all the high frequency and low frequency noise components have been removed successfully, but all the noise in the range of the bird’s song frequency (visible as grey background) is still there, confirming that this basic filtering is not sufficient to recover birdsong. Further, birds have different call categories from different frequency bands. For example, the kakapo (*Strigops habroptilus*) generate two types of vocalisation: *booming*, which is a very low frequency call and *chinging*, which is a relatively high frequency call. Designing a common filter to clean hours of kakapo recordings is impossible because they do not share the same frequency range.

Another traditional approach is the Wiener filter, which generates an estimate of the desired or target random (Gaussian) process based on linear time-invariant filtering and the minimum mean square error between the estimated signal and the desired signal by assuming that the signal and noise are stationary and spectral information is available [14]. This is not true for birdsong, therefore we did not consider it further here.

## Wavelets

We explained earlier that the Fourier transform, while commonly used in birdsong analysis, is not really suitable because of the tradeoff between temporal resolution and frequency resolution. An alternative is the *wavelet transform*, which is a relatively recent development in signal processing [15], although it has been invented independently in fields as diverse as mathematics, quantum analysis and in electrical engineering [16]. Wavelets have been applied in many areas, such as data compression, feature detection and denoising signals [17].

**Fig 4:**
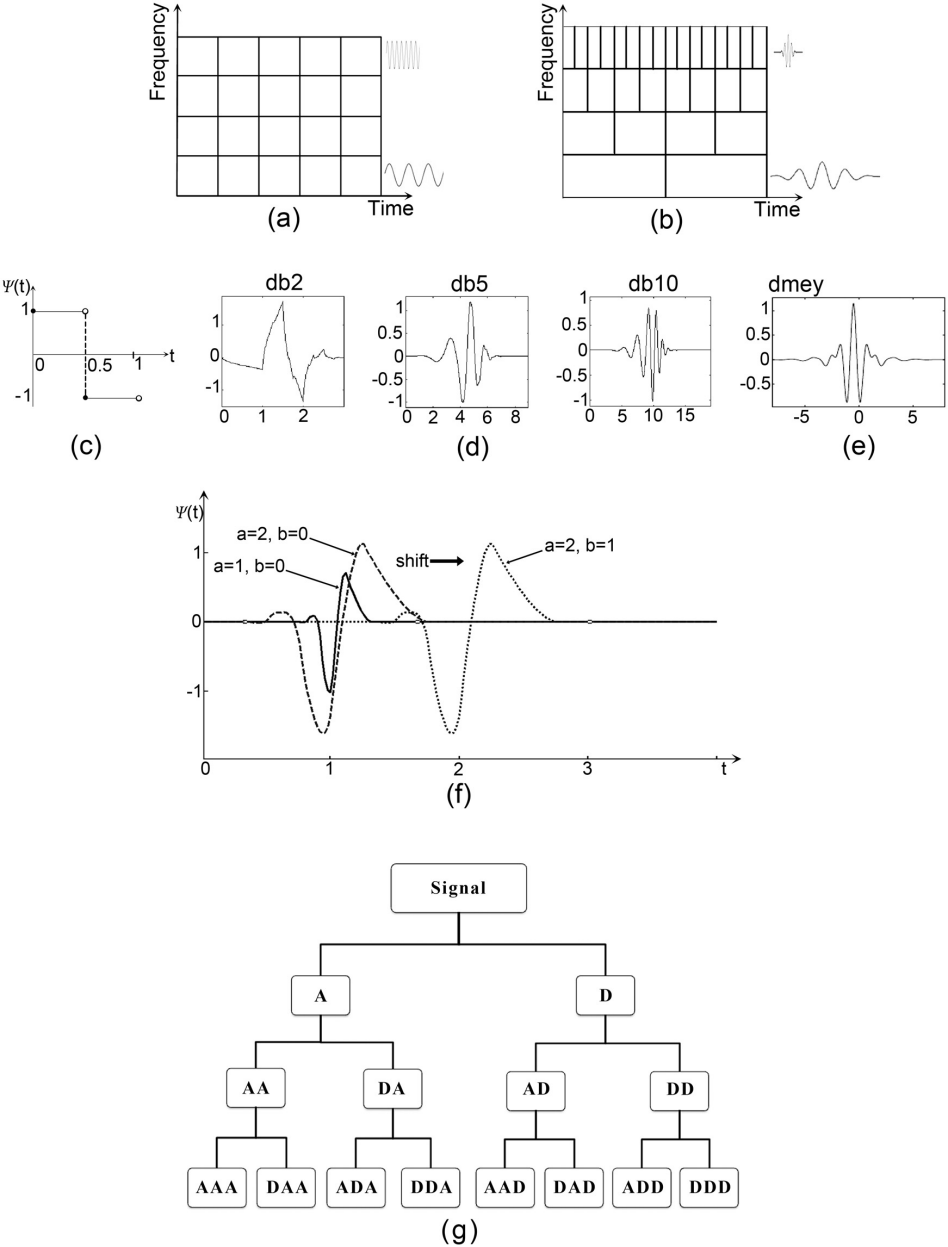
Wavelets and their relation to time-frequency resolution and wavelet packet decomposition. Time-frequency resolution in (a) STFT and (b) wavelets. Examples of mother wavelets: (c) *Haar*; (d) a subset of Daubechies wavelets; (e) Discrete Meyer wavelet. (f) Scaling and shifting the mother wavelet *Ψ*_1,0(*t*)_ gives two new wavelets *Ψ*_2,0(*t*)_ and *Ψ*_2,1(*t*)_. (g) A level three wavelet packet decomposition tree (A- approximation and D- detail).

In the Fourier transform the signal is mapped into a basis of sine and cosine waves. The wavelet transform also uses a basis, but the basis elements are scale-invariant, meaning that they look the same at all scales, and they are localised in space. The upshot is that in the wavelet representation different window sizes can be used to see the signal at different resolutions; an analogy would be viewing a forest and its trees at the same time. If we need to see the whole forest we have to see it at a large scale and then we can capture global features. In order to see the trees, we have to zoom in and to focus on a tree. Zooming more allows us to see leaves. We can see the forest, trees and even leaves by using different scales. Fig 4(a) and 4(b) highlights the difference between Fourier and wavelet analysis: the window size in Fig 4(b) is more flexible (allowing large windows for low frequencies and small windows for higher frequencies), which is important for broad spectrum non-stationary signals such as birdsong.

There are several choices of basis features (referred to as *mother wavelets*) *Ψ*, and unfortunately the best mother wavelet for a particular application needs to be determined experimentally. Fig 4(c)–4(e) shows examples of some mother wavelets, including the simplest Haar wavelet, which is a discontinuous step function. While the discontinuity can be a disadvantage in some domains, including birdsong, it is beneficial for those that exhibit sudden transitions like machine failure [18]. Fig 4(d) provides three examples of the Daubechies wavelets (*dbN*) showing that the smoothness of the wavelets increases as *N* increases. Finally Fig 4(e) shows the discrete Meyer wavelet (*dmey*).

In order to construct other elements of the wavelet basis the mother wavelet is scaled and translated by factors *a* and *b* using:

$$\Psi {}_{a,b}\left(t\right)=\frac{1}{\sqrt{\left|a\right|}}\Psi \left(\frac{t-b}{a}\right)$$ (1)

Parameter *a* ≠ 0 determines the amount of stretching or compression of the mother wavelet (depending whether *a* is greater than or less than 1). Therefore, when *a* is small high frequency components are introduced to the wavelet family; in return those wavelets can capture high frequencies of the signals. In the same manner, when *a* is large low frequency components are introduced to the wavelet family and help to capture low frequency signals. Parameter *b* determines the amount of shifting of the wavelet along the horizontal axis: *b* > 1 shifts the wavelet to the right, while *b* < 1 shifts it to the left. Therefore, parameter *b* specifies the onset of that wavelet. Fig 4(f) illustrates the effect of *a* and *b* with respect to a given mother wavelet. Accordingly, wavelets are defined by the wavelet function (*mother wavelet*) and scaling function (also called the *father wavelet*). The scaled wavelets are known as *daughter wavelets*.

### Wavelet Packet Decomposition

When wavelets are applied to a discrete signal, low-pass and high-pass filters are used, splitting the data into a low frequency (approximation) part and a high frequency (detail) part. These filtered representations of the data can then be analysed again by a wavelet with smaller scale by creating a new daughter wavelet, typically at half the scale. One modelling choice that can be made is whether to reanalyse both the approximation and detail parts of the signal, or just the approximation coefficients. We choose to analyse both, in what is known as the wavelet packet decomposition [19]. It leads to a tree of wavelet decompositions, as shown in Fig 4(g), and provides a rich spectral analysis, since there are 2^*N*^ leaves at the base of the tree when there are *N* levels.

However, the question of how many levels to use in the tree still remains. This question is often answered experimentally, but since we want a method that can work unaided on birdsong, we need to find a computational approach. We have approached this by considering how much information about the signal is contained in the approximation at each node, reasoning that nodes that do not contain information are representing the noise, and so should be discarded. In the field of information theory, Shannon entropy provides the standard measure of uncertainty or disorder in a system [20], and this is connected to the amount of information contained in a given signal [21].

The entropy *S* of a set of probabilities *p*_*i*_ is calculated as (using the convention that 0 log 0 = 0):

$$H\left(p\right)=-\sum_{i}^{}p_{i}\log_{2}p_{i}$$ (2)

where *p*_*i*_ is the probability of *i*^*th*^ state in the state space. In wavelets, we used a slightly different version of this Shannon entropy:

$$S=-\sum_{i}^{}s_{i}^{2}\ln\left(s_{i}^{2}\right)$$ (3)

where *s*_*i*_ is *i*^*th*^ sample of the signal [22, 23].

The idea of using entropy for wavelets is to argue that when the entropy is small, the accuracy of the selected wavelet basis is higher [23]. We used this computation at each node to choose whether or not to retain a node, and stopped creating the tree at the point where all of the nodes contained noise are removed by this computation, meaning that the signal was fully described.

### Previous Uses of Wavelets for Bioacoustic Denoising

The use of wavelets for noise reduction, referred to as denoising, is still an emerging advance in digital signal processing. While there are some examples of denoising in other audio signal domains such as partial discharges (PD) signals [24–26], music [27], speech [28], and phonocardiography [29, 30], their use in bioacoustic denoising is still uncommon. In addition, the two studies we know of which used wavelets for denoising animal sounds did not use natural noise, but added manual noise to their recordings. [31] denoised West Indian manatee (*Trichechus manatus latirostris*) vocalisations with added boat noise, while [32] attempted to denoise vocalizations of the ortolan bunting (*Emberiza hortulana*), rhesus monkey (*Macaca mulatta*), and humpback whale (*Megaptera novaeanglia*), with added white noise.

However, wavelets have been used for birdsong recognition: Selin et al. [33, 34] used the wavelet packet decomposition to extract features from birdsongs from eight species. Interestingly, in [34] they added noise filtering via either a low pass filter or an adaptive filter bank with eight uniformly spaced frequency bands. These filtered signals were also analysed by wavelets and compared for recognition accuracy with the unfiltered version. In addition, Chou et al. [35] used wavelets to represent birdsong in conjunction with Mel Frequency Cepstral Coefficients (MFCCs) for recognition of 420 bird species; but the dataset in their experiment was very limited, with only one recording per species (half of each birdsong file for training and the remaining for testing).

### Our Algorithm

To summarise our approach to birdsong denoising, we took the following steps, which are discussed further next:

1. Find a suitable mother wavelet.
2. Find the most suitable decomposition level based on the Shannon entropy.
3. Apply the wavelet transform to the noisy signal to produce the noisy wavelet coefficients.
4. Determine the appropriate threshold to best remove the noise based on the Shannon entropy.
5. Invert the wavelet transform of the retained wavelet coefficients to obtain the denoised signal.
6. Apply a suitable ordinary band-pass or low-pass filter where possible to remove any noise left outside the frequency range of the signal.

#### Selecting the Mother Wavelet

Choosing an appropriate mother wavelet is the key to the successful estimation of the noiseless signal. One approach is to visually compare the shapes of the mother wavelets and small portions of the signal, choosing the wavelet that best matches the signal [25]. However, given that we want the method to work with a wide variety of different bird calls, eyeball selection is not sufficient.

**Fig 5:**
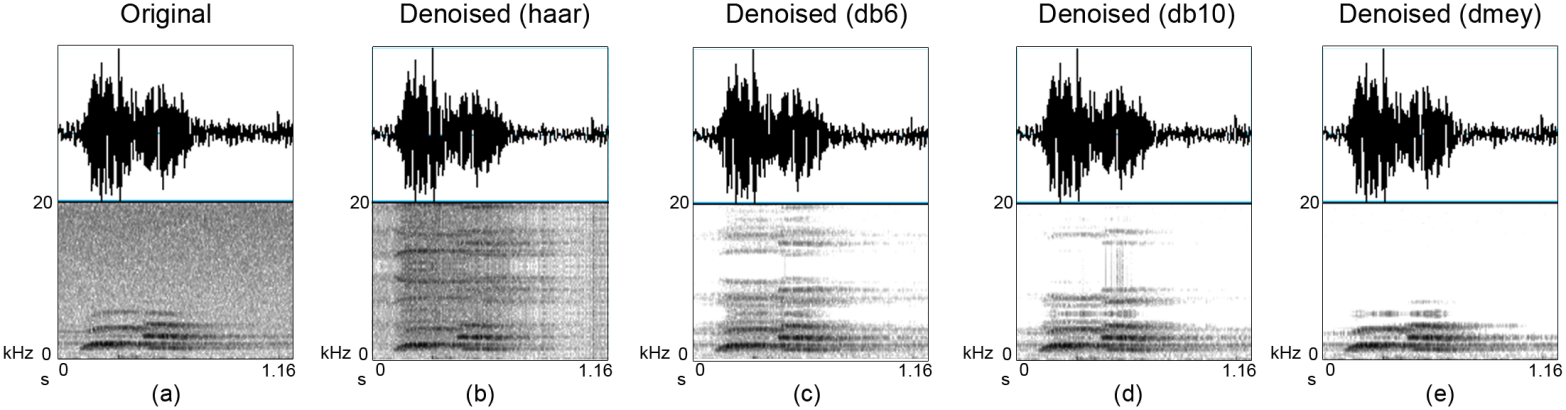
Different mother wavelets produce different results. Same excerpt of a male kiwi whistle (a) original whistle and (b)—(e) denoised with different mother wavelets.

Another approach is based on the correlation between the given signal and its denoised signal [36], reasoning that if two signals are strongly linked they should have high correlation. Therefore, we can expect that the optimum wavelet maximises the correlation of initial signal and denoised signal. We can compare the correlation under different wavelets and pick the wavelet that generates the highest correlation. Accordingly, we analysed the correlation given by different wavelets including the Daubechies wavelets (*dbN*, where *N* represents the order) and the Discrete Meyer wavelet (*dmey*). Initial experiments showed that the *dmey* wavelet generated the highest correlation. For instance, *db*2 (0.9950) was better than *db*1 (0.9884), *db*6 (0.9970) was better than *db*2, *db*10 (0.9971) was better than *db*6, and *dmey* (0.9973) was better than *db*10. Then, we investigated the spectrograms of the denoised examples in order to see the actual improvement of the songs. Visual inspection (for example Fig 5) also confirmed that the *dmey* wavelet (Fig 4(e)), successfully denoised the songs without distorting them with a selection of different birdsongs, and so we used that for the rest of our experiments.

#### Selecting the Best Decomposition Level

Because we used Shannon entropy to choose the decomposition level, different birdsongs will produce trees of different depths: less complex birdsong will have small trees, while more complex birdsong will require larger trees. In fact, even within single types of call, different depths of tree can be seen. We therefore ran the depth selection algorithm on every birdsong individually; while this is computationally expensive, it does lead to significantly better results. Methods to speed up this approach will be investigated in future work. So far we found that the top-down approach (start with a small tree with level 1 and expand it based on the Shannon entropy) is more efficient than the bottom-up approach (start with a big tree and shrink it); therefore we used the top-down calculation here. Starting from level 1, decomposition was continued until the maximum entropy of a parent node (at level *L*) was lower than the maximum entropy of its child nodes (at level *L*+1). At that point the decomposition was stopped, and the best decomposition level was determined as *L*.

#### Selecting the Threshold

Each node in the decomposition tree is represented by its wavelet coefficients, and the ‘impurity’ of those nodes can be calculated using (Shannon) entropy. Then, eliminating noisy nodes is done by applying a threshold to each node. There are two forms of thresholding methods: *hard thresholding* and *soft thresholding*. In hard thresholding, sometimes called the ‘keep or kill’ method [37], coefficients are removed if they are below a previously defined threshold. In contrast, soft thresholding shrinks the wavelet coefficients below the threshold rather than cutting them off sharply. Soft thresholding provides a continuous mapping and in our case it demonstrated better noise reduction without information loss yielding high SnNR (this term will be defined in the section on evaluation metrics) in initial experiments. Therefore, we used soft thresholding here.

The challenge of setting the threshold remains, however: ideally, the selected threshold should achieve satisfactory noise removal without significant information loss. If the selected threshold is too high, then it removes too many nodes from the tree, resulting in a denoised signal with missing information, while if the threshold is unnecessarily low, it does not remove all the noisy nodes, resulting in a signal that still has noise in. There will be no globally optimal threshold, and so we again selected it based on analysis of each birdsong. As was mentioned previously, many types of noise can be approximated as having a Gaussian distribution, and this is more obvious in the high frequency parts of the spectrum. We therefore computed the standard deviation of the lowest level detail coefficients in the tree, and used 4.5 standard deviations as the threshold, which should cover 99.99% of the noise [26].

## Experimental Evaluation

In this section we compare our wavelet-based algorithm with traditional band-pass or low-pass filtering. We introduce our dataset, and the metrics that we use to compare the results, before demonstrating the results.

### Datasets

#### Primary Dataset

Initially, three manually generated pure sound examples (one impulsive ‘click’ sound and two tonal combinations) were used to examine the performance of the proposed method against white and different coloured noises. These examples were separately polluted with different levels of these noises manually and then denoised to eliminate the noise.

**Fig 6:**
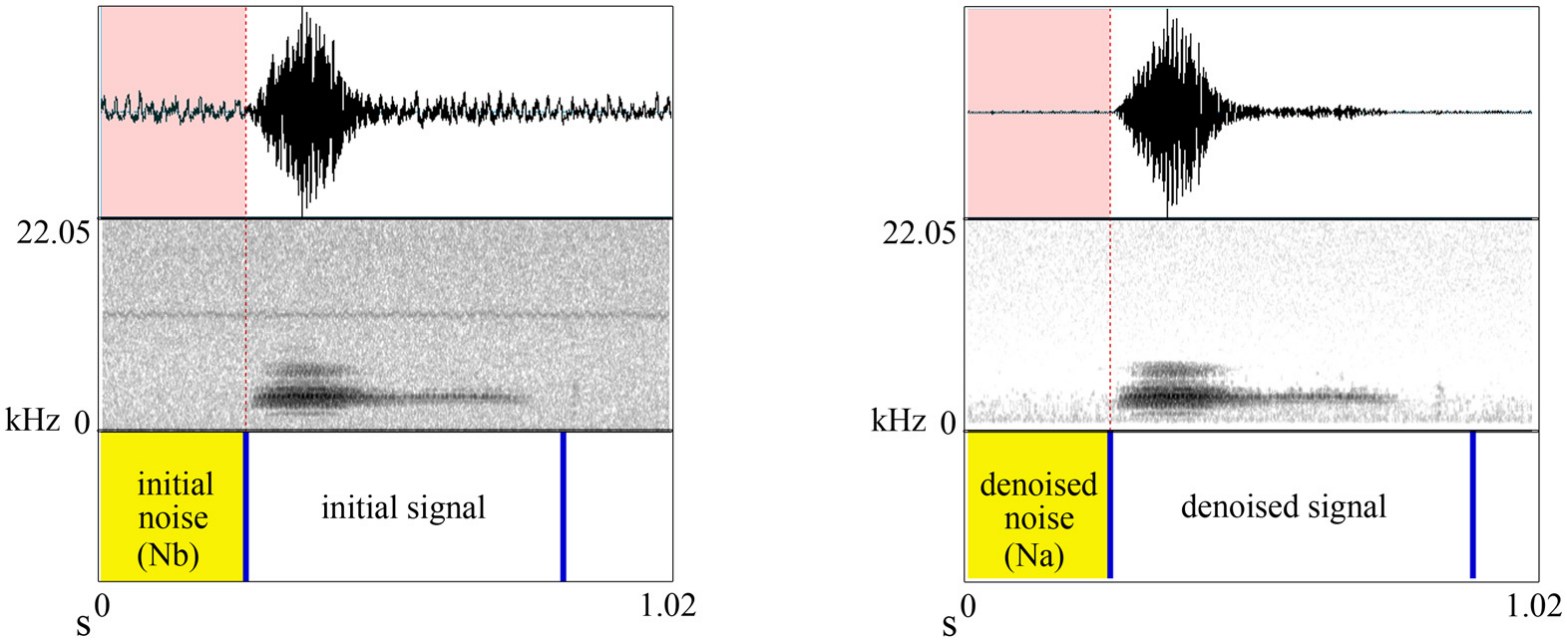
An example of kakapo *chinging* used in the experiment. Top, middle, and last rows represent oscillogram, spectrogram, and labels indicating the parts of the recording used to calculate the SnNR respectively. (a) Initial signal and (b) the same signal after denoising and band-pass filtering.

Secondly, songs of two endangered and one relatively common New Zealand bird species were considered: North Island brown kiwi, kakapo, and ruru (*Ninox novaeseelandiae*). Most of the recordings were collected using automated recorders, but a few were recorded manually. Most ruru and kiwi calls were obtained by the authors, while some ruru and all kakapo calls came from other sources (see the Acknowledgements). The spectrogram patterns of these species are shown in Fig 1. Birdsongs were segmented manually into syllable level components (e.g., Fig 6). The dataset (available at http://avianz.massey.ac.nz) contained a total of 700 syllables from seven basic call types, 100 of each (Table 1). These recordings were polluted with different types and levels of noise while recording. Mainly the noise was wide-band; sometimes it was concentrated more to low frequencies (for example due to wind and aeroplane noise) others to high frequencies and/or to narrow bands (for example due to insect noises like crickets and weta).

**Table 1:** List of species, their call types and frequency range.

Species/call type	Observed frequency range (Hz)
North Island brown kiwi	
*male*	500–8,000
*female*	500–6,500
Ruru	
*trill*	500–8,000
*more*	500–2,000
*pork*	500–2,000
Kakapo	
*booming*	0–800
*chinging*	1,000–12,000

We use the common names given by researchers for the different types of calls.

#### Secondary Dataset

We tested our algorithm on a secondary data set because our primary data set did not cover all possible spectrogram patterns we expect to see in recordings collected in the wild. The songs in this data set were mostly collected by the authors using manual recorders, but the selected recordings include significant amounts of noise. The kaka and tui songs were recorded using automated recorders by others. The eight species in this dataset (see Table 2) comprise seven song birds and one parrot, which have complex songs and great song diversity. We used whole songs instead of syllable level components. Five noisy song examples of each species were used, except for hihi; this species has very short songs and therefore we used ten examples.

**Table 2:** List of species introduced to the secondary dataset and their song characteristics.

Common name	Scientific name	Observed frequency range (Hz)	Song structure
North Island robin	*Petroica longipes*	1,700–12,500	Males sing loud songs that have series of phrases. Phrases have variety of simple notes.
Tui	*Prosthemadera novaeseelandiae*	400–18,000	Loud and complex songs: mix of melodious notes with coughs, grunts and wheezes.
North Island kaka	*Nestor meridionalis*	700–15,000	Harsh and grating sound, variety of musical whistles.
Hihi	*Notiomystis cincta*	1,000–21,000	Variety of 2–3 note whistles. Quiet or aggressive warbles.
North Island saddleback	*Philesturnus rufusater*	800–22,000	Very active and noisy. Loud chattering calls and variety of rhythmical songs.
Marsh wren	*Cistothorus palustris*	500–15,000	Gurgling and rattling trill.
Western meadowlark	*Sturnella neglecta*	650–12,500	Male sings a complex, two-phrase song, begins with 1–6 pure whistles then a series of 1–5 gurgling warbles.
Horned Lark	*Eremophila alpestris*	1,100–18,000	Musical songs: fast, high-pitched sequence of sharp, tinkling notes.

Further, we were interested to see the performance of this technique over unsegmented recordings. Therefore, we denoised five series of consecutive calls from each call type from each species mentioned in the primary data set. Then we compared the calls in the denoised series to their respective segmented calls.

Another concern when denoising birdsong is the effect of overlapping bird calls. To test this issue, we selected ten examples of recodings that contained overlapping songs from different combinations of species. Examples include overlapped male kiwi-female kiwi, male kiwi- ruru trill, male kiwi-more-pork, two of more-pork-trill, two of male kiwi-female kiwi-more-pork, tui-more-pork, robin-tui, and kakapo chinging-mottled petrels. Again the dataset is available at http://avianz.massey.ac.nz.

### Evaluation Metrics

The main measurement of true interest in denoising is the Signal-to-Noise Ratio (SNR), which can be calculated by dividing the power of the signal (*S*) by the power of noise (*N*), as given in Eq 4, which is in units of decibels (dB). The higher the value of the SNR, the less noisy the signal.

$$SNR=10\log_{10}\left(\frac{S}{N}\right)$$ (4)

**Fig 7:**
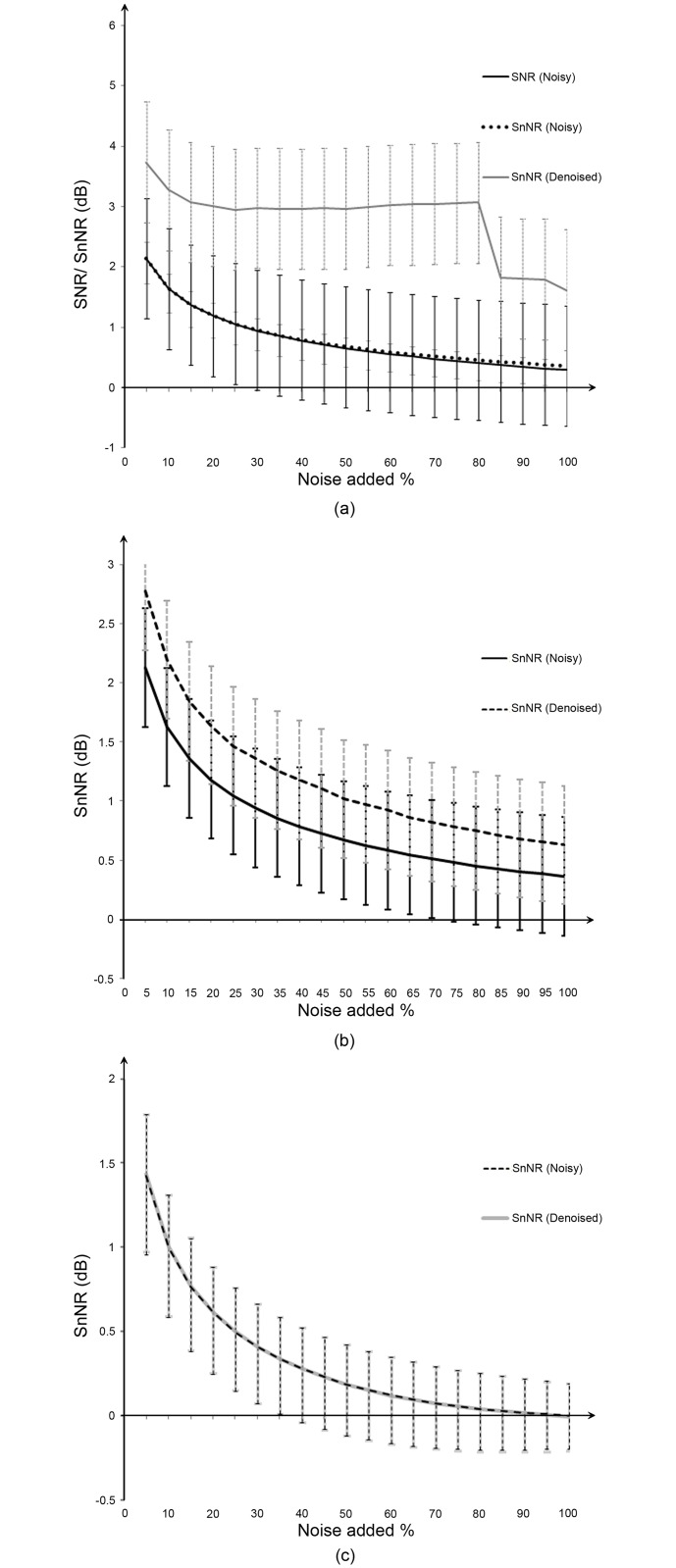
Denoising different types of noise. (a) White noise, (b) pink noise, and (c) brown noise.

The challenge for real-world applications such as birdsong is that the signal and noise are not actually known because they are together in the recording. This means that computing *S* and *N* is not actually possible. Under the assumption that the noise is relatively stationary, we have estimated the power of the pure noise by isolating parts of the recording without birdsong, which should theoretically be silent, and modified Eq 4:

$$SnNR=10\log_{10}\left(\frac{S+N}{N}\right),$$ (5)

where *S* + *N* is the power in the initial signal. By comparing this computation with the denoised version we can see how effective the denoising is. Fig 6 illustrates the calculation. Notice that to be able to calculate the initial noise and denoised noise we segmented the recording leaving a small period of silence at the beginning and/or end of the bird call. A comparison of original SNR and respective SnNR are shown in Fig 7, where noise and signal are known.

If we recall that the noise is approximately Gaussian, a second possible metric is to measure its statistical properties, particularly its variance, reasoning that successful denoising should substantially reduce the variance of the noise. We used the same segments of ‘pure’ noise in the signal as were used to estimate the power of the noise in the SnNR to compute the variance of the noise before and after denoising, terming this measure the *success ratio*:

$$\text{Success}\;\text{ratio}=\log_{10}\left(\frac{\text{var}(Nb)}{\text{var}(Na)}\right),$$ (6)

where *Nb* is the initial noise and *Na* is the noise after denoising. If the success ratio is greater than 0, it implies that song denoising has been successful.

A third possibility is to calculate the Peak Signal to Noise Ratio (PSNR), a widely used objective quality metric in image and video processing [38, 39]. PSNR looks only at the peak value the signal can reach and the mean-squared error between the reference and the test signals. Here we used a modified PSNR [40] to compare noise reduced songs with their original noisy version.

$$PSNR=10\log_{10}\left(\frac{MAX_{sig}^{2}}{MSE}\right)=20\log_{10}\left(\frac{MAX_{sig}}{\sqrt{MSE}}\right),$$ (7)

where *MAX*_*sig*_ is the maximum value of the reference signal and MSE is the mean-squared error. In this calculation we maintained the noisy song as the reference and its recovered song as the test. PSNR will be relatively lower if the song is less cleaned and higher if the song is well cleaned.

## Results

We implemented our algorithm in Matlab using the Wavelet Toolbox, which is a comprehensive toolbox for wavelet analysis. The code is available at: http://avianz.massey.ac.nz. As an initial experiment, white noise, pink noise, and brown noise were added to selected tonal and impulse sounds separately as a percentage of the strength of the signal. These noisy examples were cleaned using the proposed denoising approach (steps 1–5 only; without filtering), and the calculated SNR and SnNR of noisy and recovered songs are plotted in Fig 7. Here we can calculate the SNR of the noisy examples perfectly because we know the actual noise added as well as the pure signal. A comparison of conventional SNR and SnNR is illustrated in Fig 7(a) confirming that both metrics perform almost equally. The same figure also shows that even in the presence of high levels of white noise, denoising using our approach is very successful. Fig 7(b) reveals that the proposed denoising approach can deal well with pink noise, but not to the extent of white noise. However, denoising brown noise still remains a challenge as shown in the Fig 7(c). This is because of its strong non-Gaussianity.

**Fig 8:**
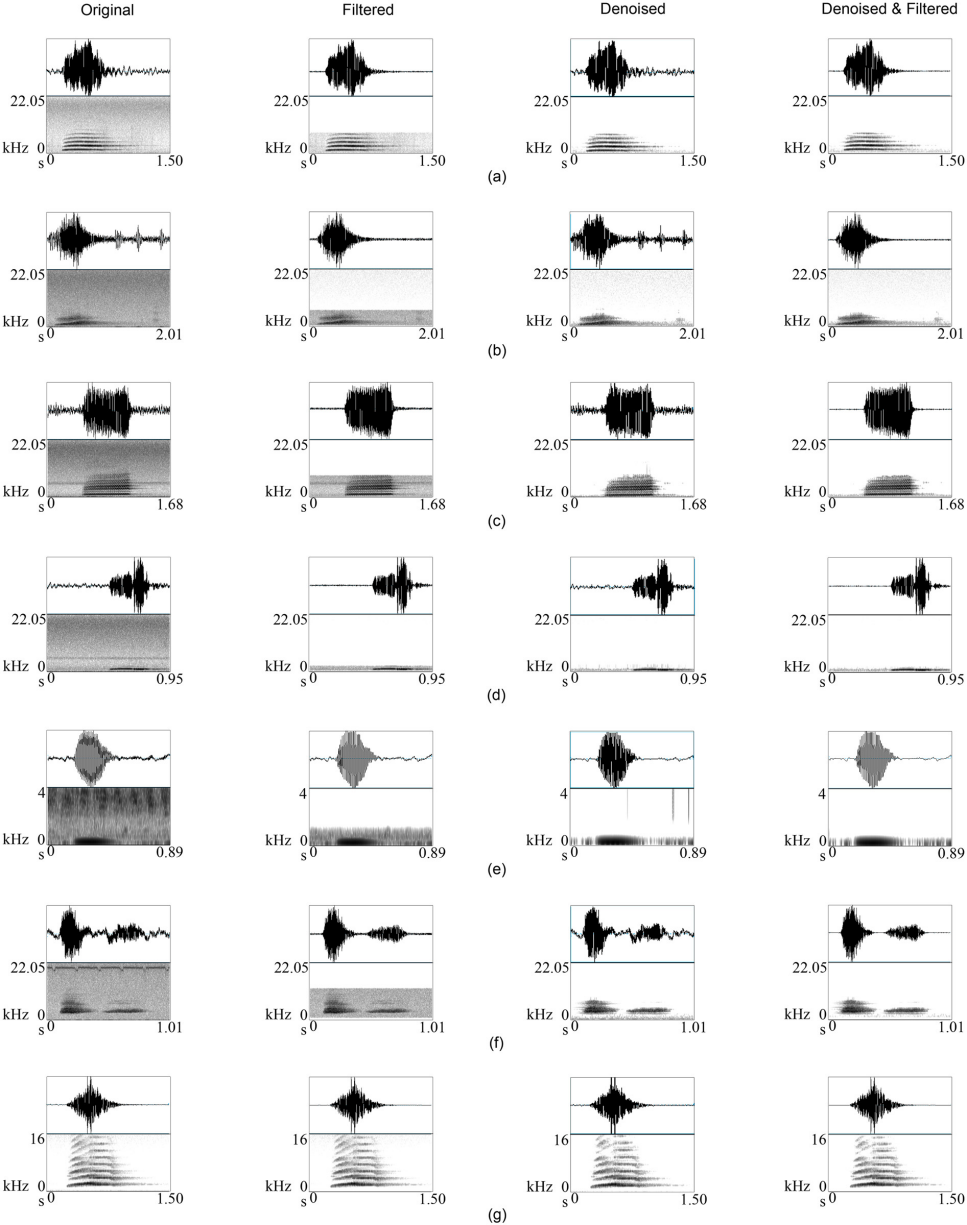
Bird call examples of before, after filtering, after denoising using wavelets as described in the text, and after denoising and classical filtering. (a) A whistle of a male North Island brown kiwi, (b) a call of female North Island brown kiwi, (c) a ruru *trill*, (d) a ruru *more*, (e) a kakapo *booming*, (f) a kakapo *chinging*, and (g) a less noisy example of male kiwi.

**Fig 9:**
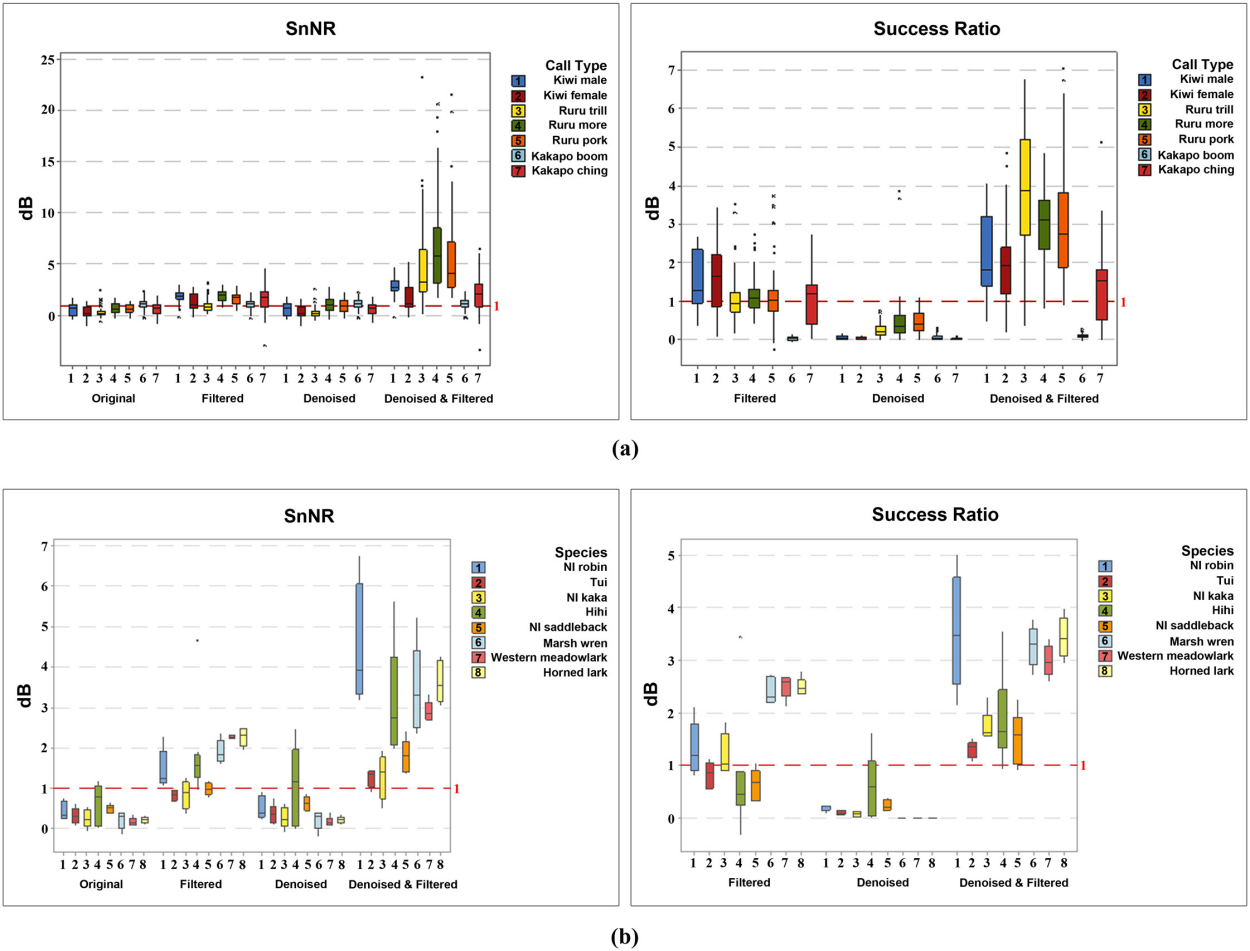
Box plot view of the results in (a) Table 3 and (b) Table 4.

Each call example in both primary and secondary datasets was treated with three approaches: band-pass or low-pass filtering alone (F), wavelets alone (D), and wavelets and band-pass or low-pass filtering (DF). In the case of filtering, the frequency bands were selected according to Tables 1 and 2. Fig 8 (S1 Audio) demonstrates that our algorithm removed most of the noise from the birdsong while preserving most of the song information. Success is visually clear from the spectrograms, for example if we consider Fig 8(a), almost all the background grey colour (caused by noise) in the original kiwi whistle has been eliminated, while the five original harmonics are still present without distortion after denoising. We examined visually and aurally each example individually to confirm whether they were improved after denoising, and found that all the calls were significantly improved. The improvement in the sound quality of the songs was successfully reflected by SnNR and Sussess Ratio (Table 3 and Fig 9). The overall SnNR improved from 0.667 to 3.506, an improvement of more than 5 times while SnNR improved only up to 1.526 after conventional filtering. Success ratios after filtering alone and with denoising were 1.071 and 2.170 respectively. Parallel to this, PSNR increased from 10.428 to 10.694. While we have included PSNR (Eq 7) in our results, we do not believe that it is a particularly useful measure. First, the numerator uses the maximum amplitude (hence the ‘peak’ in the name), which does not change when the signal is denoised, while the denominator is the root mean square of the error, which is small. This leads to an less sensitive measurement. For example, denoising alone always generated the highest PSNR because the oscillogram was not substantially changed after denoising as much as it does with filtering (see Fig 8) leading to a comparatively small MSE. However, these results altogether confirm that our wavelet denoising approach performs really well for birdsong. Even for the very low frequency kakapo *booming* the denoising was still better with wavelets. On the other hand, in the case of less noisy bird calls, after denoising there was no significant information loss (see Fig 8(g)).

**Table 3:** Experimental Results—primary data set.

Species/		O	F			D			DF			WMDL
call type		SnNR	SnNR	S ratio	PSNR	SnNR	S ratio	PSNR	SnNR	S ratio	PSNR	
Kiwi	male	0.666	1.918	1.509	10.849	0.710	0.063	36.145	2.877	2.156	10.935	10
	female	0.405	1.379	1.536	11.860	0.423	0.035	39.633	1.690	1.801	11.942	10
Ruru	trill	0.341	0.988	1.075	10.902	0.365	0.261	22.569	4.792	3.840	11.695	10
	more	0.761	1.940	1.170	10.596	1.121	0.482	25.758	6.463	2.979	10.963	8
	pork	0.676	1.702	1.114	9.159	1.034	0.457	24.852	5.520	2.950	9.574	8
Kakapo	boom	1.136	1.138	0.032	4.843	1.187	0.080	43.554	1.184	0.105	4.889	6
	ching	0.682	0.617	1.059	14.790	0.703	0.036	45.416	2.016	1.360	14.857	9
**Total/mean**		**0.667**	**1.526**	**1.071**	**10.428**	**0.792**	**0.202**	**33.990**	**3.506**	**2.170**	**10.694**	**9**

O = original calls. F = band-pass or low-pass filtered calls. D = wavelet denoised calls. DF = wavelet denoised and filtered calls. S ratio = success ratio, SnNR = Signal to Noise Ratio and PSNR = Peak Signal to Noise Ratio introduced in Evaluation Metrics. WMDL = Wavelet Mean Decomposition Level.

As discussed under Selecting the Best Decomposition Level, our automated method selects the appropriate decomposition level based on the complexity of the given signal. We classified the complexity of a song by examining the spectrogram pattern and listening to the sound; songs with more harmonics and wide frequency range were considered more complex (for example, in the case of ruru, trill calls are rather complex compared to narrow band more-pork calls). The results confirm that there is a relationship between the best decomposition level and the complexity of birdsongs: if we order the calls according to their complexity from simplest to complex, the order is kakapo *booming*, *more* and *pork*, kakapo *chinging*, kiwi *female*, kiwi *male*, and finally ruru *trilling*, and this order can be seen in the depth of the tree (WMDL) in the last column of Table 3.

### Extensions

**Fig 10:**
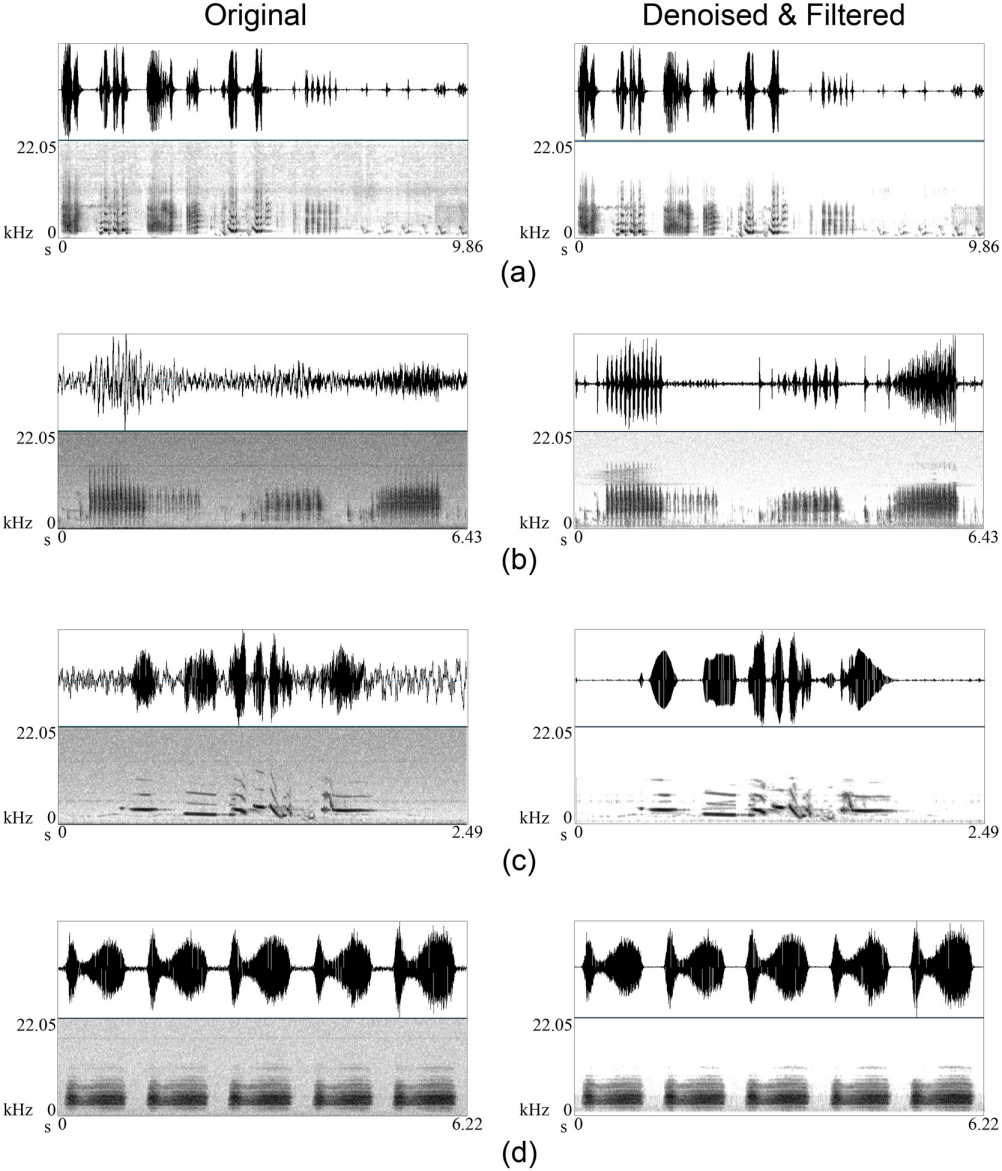
Denoising entire songs and long series of calls. (a) A North Island kaka song, (b) a marsh wren song, (c) a western meadowlark song, and (d) a series of kakapo *chinging*.

Our method achieved impressive noise removal for the birdsongs of the species we considered in the secondary data set. Table 4 and Fig 9 show that the overall SnNR reached more than seven fold (2.758) after the treatment compared to their initial SnNR (0.353). Some examples of these songs are presented in Fig 10 (S2 Audio).

**Table 4:** Experimental Results for the species introduced to the secondary data set.

Species	O	F			D			DF		
	SnNR	SnNR	S ratio	PSNR	SnNR	S ratio	PSNR	SnNR	S ratio	PSNR
NI Robin	0.461	1.464	1.324	16.907	0.530	0.202	30.784	4.534	3.552	17.393
Tui	0.320	0.840	0.830	13.977	0.369	0.114	31.907	1.259	1.313	14.218
NI kaka	0.258	0.850	1.214	15.714	0.282	0.085	34.644	1.296	1.747	15.939
Hihi	0.651	1.814	0.745	11.446	1.133	0.692	29.259	3.217	1.884	11.794
NI saddleback	0.505	0.987	0.636	14.563	0.631	0.254	28.981	1.783	1.508	15.038
Marsh wren	0.220	1.910	2.426	16.511	0.219	0.011	41.837	3.423	3.280	16.579
Western meadowlark	0.180	2.277	2.520	12.437	0.181	0.011	41.807	2.906	2.994	12.474
Horned lark	0.225	2.271	2.491	12.356	0.228	0.017	39.846	3.647	3.448	12.402
**Mean**	**0.353**	**1.552**	**1.523**	**14.239**	**0.447**	**0.173**	**34.883**	**2.758**	**2.466**	**14.480**

**Fig 11:**
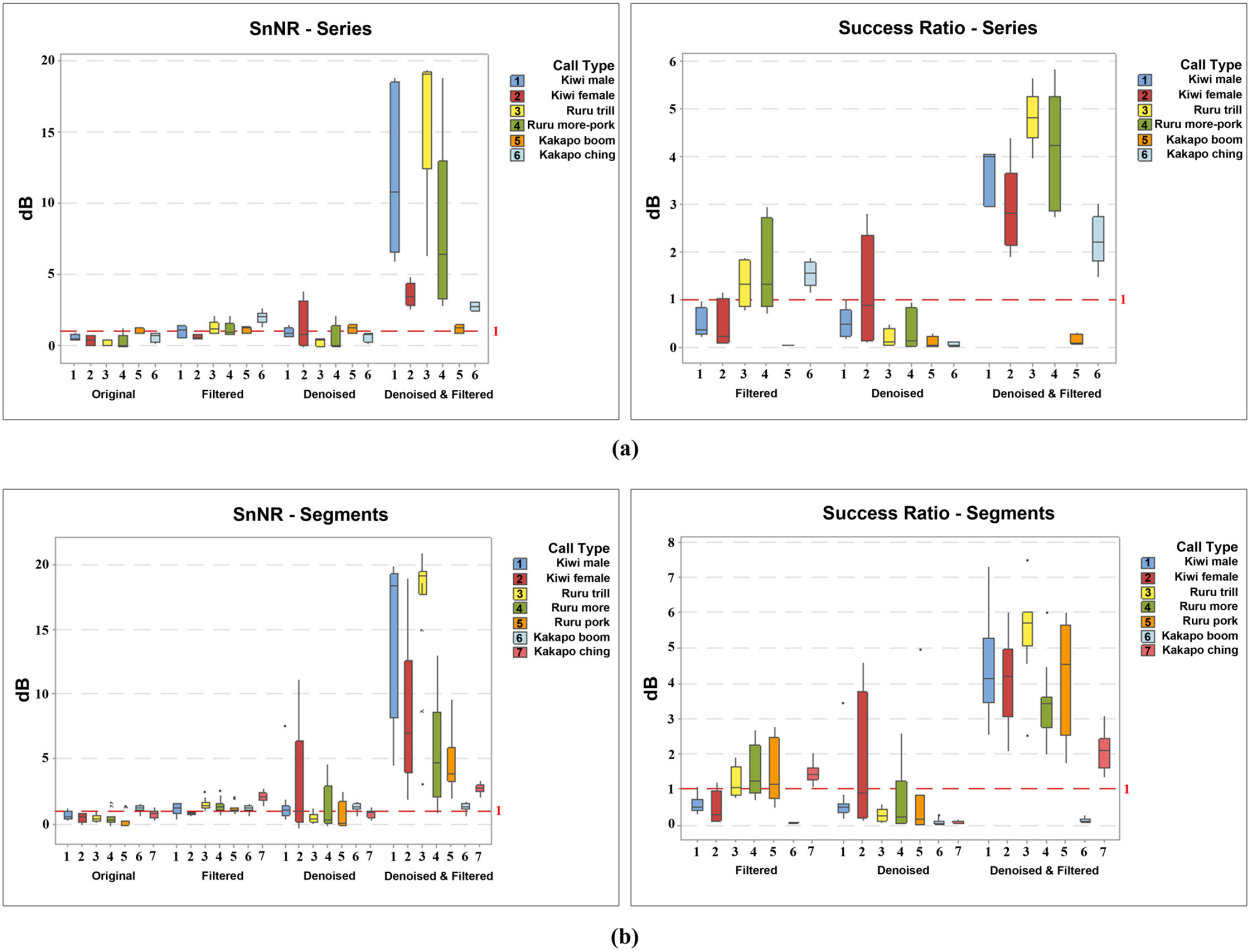
Box plot view of Table 5. (a) call series and (b) segmented calls.

While the main aim of our approach was to denoise individual bird calls, we also considered two extensions: denoising a series of bird calls in a sequence without segmenting them, and denoising a signal that is comprised of two or more overlapping bird calls. Table 5 and Fig 11 compare the results of denoising unsegmented series of bird calls to their segmented calls. In the presence of unsegmented recordings, the mean SnNR of initial, filtered, and denoised songs were 0.548, 1.204, and 7.326 respectively. This success was confirmed by further analysis of their spectrograms and sound quality, for example, Fig 10(d) shows a denoised version of a series of kakapo *chinging*. These results support the fact that denoising unsegmented long recordings is also possible and performs nearly equally to denoising their isolated calls.

**Table 5:** Comparing the denoising results—series of calls against their segmented calls.

Species/type		#	O	F			D			DF		
		e.g.	SnNR	SnNR	S ratio	PSNR	SnNR	S ratio	PSNR	SnNR	S ratio	PSNR
Kiwi (series)	male	5	0.584	1.015	0.522	13.325	0.968	0.513	23.453	12.159	3.610	14.172
	female	5	0.406	0.657	0.498	14.179	1.447	1.170	21.759	3.583	2.876	15.569
Kiwi (seg.)	male	20	0.659	1.187	0.585	11.588	1.359	0.626	20.776	14.128	4.365	12.456
	female	18	0.524	0.818	0.483	11.595	3.143	1.790	19.196	8.909	4.108	13.021
Ruru (series)	trill	5	0.272	1.269	1.334	13.647	0.288	0.207	25.504	16.453	4.828	14.337
	more-pork	5	0.290	1.143	1.689	13.125	0.566	0.375	26.587	7.782	4.088	13.386
Ruru (seg.)	trill	14	0.437	1.458	1.184	11.256	0.440	0.287	20.791	17.157	5.465	12.358
	more	11	0.464	1.413	1.449	10.337	1.367	0.716	22.830	5.547	3.433	10.689
	pork	11	0.308	1.209	1.457	9.157	0.584	0.808	20.865	4.459	4.122	10.040
Kakapo (series)	boom	5	1.118	1.129	0.059	8.308	1.202	0.120	43.865	1.207	0.170	8.332
	ching	5	0.617	2.009	1.553	13.906	0.640	0.072	39.032	2.769	2.258	14.051
Kakapo (seg.)	boom	21	1.184	1.196	0.063	4.810	1.245	0.082	43.037	1.250	0.135	4.838
	ching	17	0.768	2.069	1.467	12.286	0.802	0.076	38.627	2.709	2.062	12.426
**Mean**	**series**		**0.548**	**1.204**	**0.943**	**12.748**	**0.852**	**0.410**	**30.033**	**7.326**	**2.972**	**13.308**
**Mean**	**seg.**		**0.620**	**1.336**	**0.955**	**10.147**	**1.277**	**0.626**	**26.589**	**7.737**	**3.384**	**10.833**

**Fig 12:**
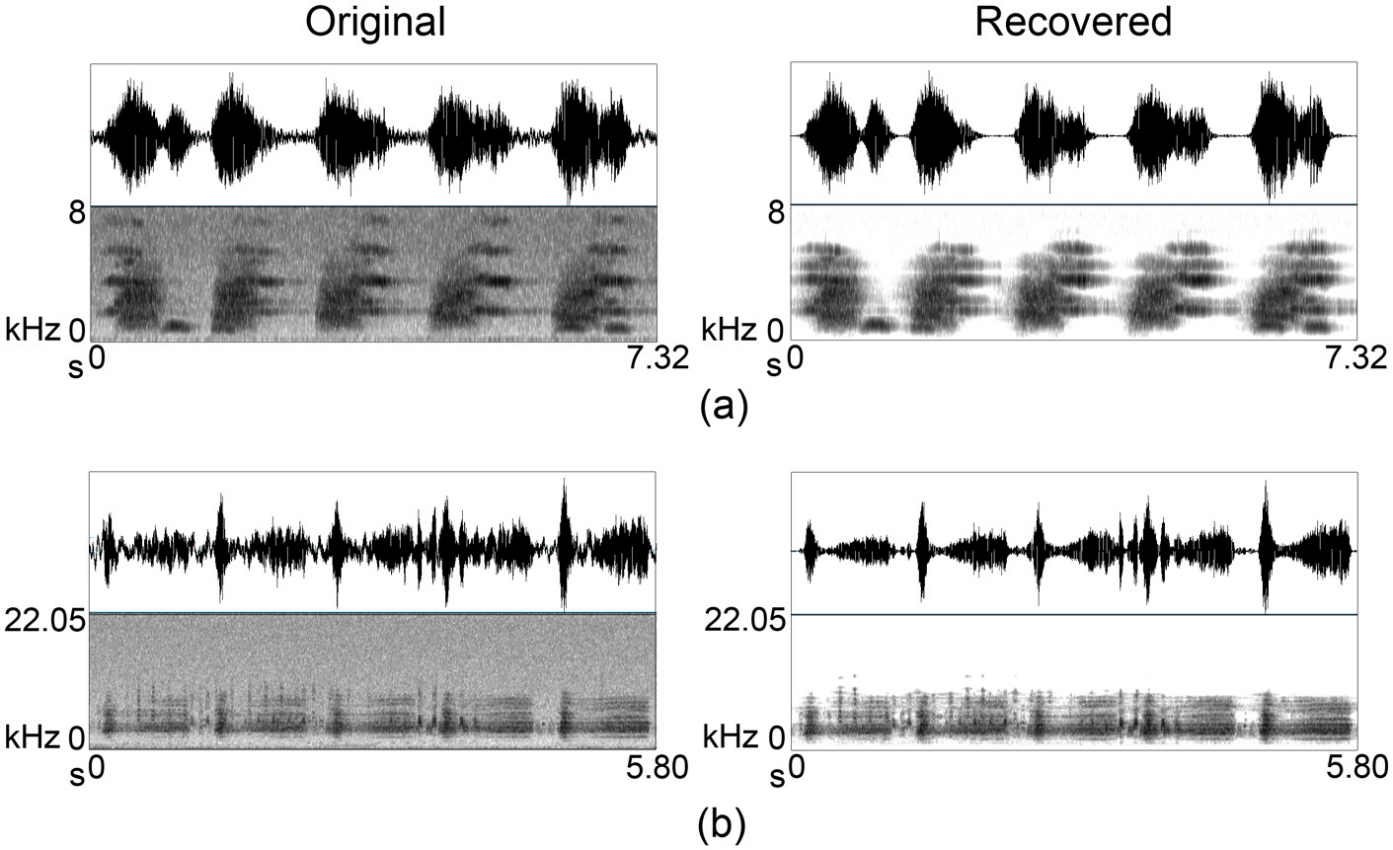
Denoising overlapped songs. Male kiwi, female kiwi, and more-pork are overlapped in (a) and kakapo *chinging* overlapped with mottled petrels (*Pterodroma inexpectata*) in (b).

The method worked very well even when presented with more than two overlapping birdsongs, with the combination of the birdsongs being retained, but the noise significantly reduced (Fig 12, S3 Audio). This is also reflected in the evaluation metrics where overall SnNR improved significantly from 0.556 to 5.222 (more than 9 times) after denoising and band-pass filtering compared to band-pass filtering alone (1.652–less than 3 times) and denoising alone (0.634) for the ten examples described at the end of the Section Secondary Dataset. Confirming the potential, success ratio displayed a significant improvement after treating the examples with denoising and band-pass filtering (2.994) than filtering alone (1.261) and denoising alone (0.169). As usual, PSNR was highest with denoising alone (28.543) while filtering (12.818) and the combination of denoising and filtering (13.205) displayed relatively low PSNR because of the increase of MSE.

## Discussion

The spectrogram has been the basis for much of birdsong analysis for decades, but it suffers from a fundamental tradeoff between temporal and frequency resolution because it is based on the Fourier transform. The more modern approach of wavelet analysis does not suffer from this tradeoff. However, there have been surprising few published studies in the field of birdsong recognition where wavelet analysis is used [32–34]

In this paper we have investigated the use of wavelets for denoising automatically recorded birdsong. Denoising is becoming progressively more important as larger numbers of automatic recorders are deployed worldwide, recording not just birdsong, but every other noise in the environment. Whether these recordings are analysed automatically or manually, there is a need to reduce the extraneous noise from the recordings. We have demonstrated that wavelets are very good at washing out stationary noise from the recordings without distorting the birdsongs. Even though some of the background noise (such as other animals) are not stationary, there is a substantial amount of stationary noise in recordings collected from nature. Therefore the applicability of this method to clean natural acoustic recordings is high. Further, much (although not all) natural noise is white or pink, and wavelets work well for removing it. Both the *success ratio* and modified SNR (SnNR) are useful measures of noise reduction. However, PSNR turned out to be a less reliable method to evaluate the success of noise reduction of audio signals.

This is one step towards our ultimate goal of automatically recognising birdsong by algorithm, and so our real aim is not the reproduction of a perfectly noiseless birdsong, but to remove the noise without damage to the signal, so that features of the song can be computed and used as input to other algorithms. In practice, the major reason for low recall rate or sensitivity (the percent of songs retrieved from the total number of songs in the recording) as well as low song recognition rate is the noise associated with the recordings: noise mixed with birdsong tends to hide song information [41, 42]. Therefore, cleaning the recordings prior to call detection and segmentation would improve any method of song recognition. However, we have demonstrated that our method also allows impressive reproduction of denoised birdsong for use by biologists. On the other hand, this reproduction capability provides more flexibility to extract any preferred features for classification and recognition in contrast to the case in [34].

**Fig 13:**
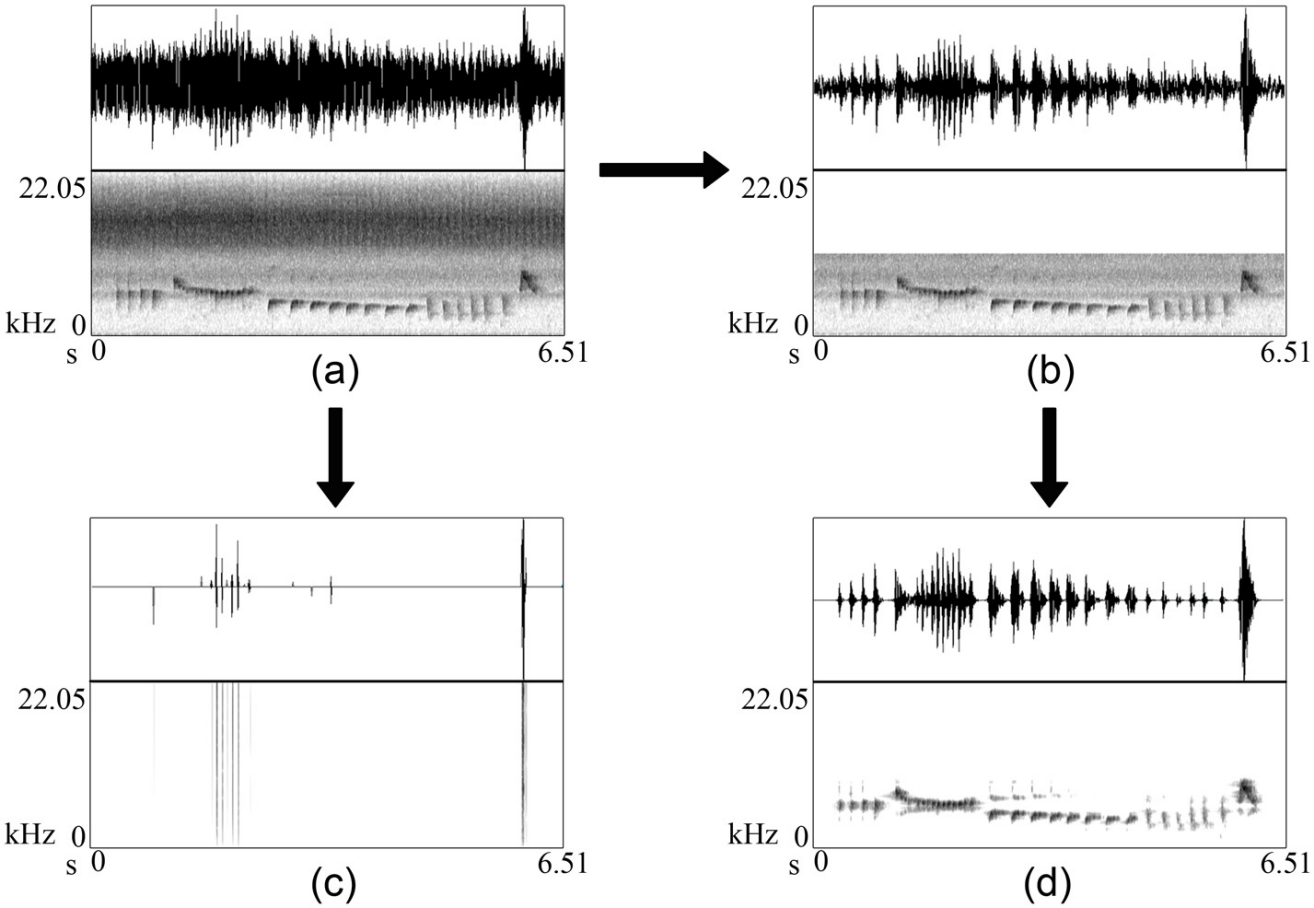
A deliberate denoising example. (a) A North Island robbin song (sampling frequency 44,100 Hz) and (b) its down-sampled song to 22,000 Hz. (c) and (d) are their denoised songs.

The major challenge of using this method to clean long field recordings is its high computational cost: it requires significant computer memory and time. The complete analysis of approximately 2 minutes of calls (the segmented versions in Table 5) took approximately just over 10 minutes on a 2.4 GHz quad core i7. This can be improved through a compiled implementation of the method, rather than the general research-focused Matlab code that we have used here. In addition, we have demonstrated our approach on fairly long recordings so that we are confident that this method can be used to clean those too. Noise removal from the original recordings rather than from extracted isolated songs is really important both in semi-manual and in automated recognition. However, it is important to note that the level of noise, its nature, and strength of the song can cause significant effect when denoising using wavelets. For example we observed that denoising tended to remove both signal and noise when presented with very faded calls embedded in a high level of noise (calls that are hardly visible in the spectrogram). We observed the same when we inputted a North Island robin song mixed with strong noise at high frequencies (Fig 13, S4 Audio). Interestingly, in this case, down-sampling saved the birdsong. If we initially apply low-pass filtering to filter out the frequencies beyond birdś frequency range, we end up with a signal that contains the birdsong and non-Gaussian noise. This means that when we filter out the high frequency noise, the signal still has capacity for high frequencies unless we down sample it. Therefore, wavelet denoising cannot remove the remaining noise because it is non-Gaussian. In contrast, down sampling restricts the signal’s frequency range, and automatically removes high frequency noise.

Generally, birds produce vocalisations within the range of human hearing. The dedicated recording devices we normally use in the field are also made to capture audible frequencies, but not ultrasonic (> 20kHz) or infra-sound (< 20Hz). However, many species produce sounds that are outside human and machine range. For example, species like the kakapo, North African Houbara bustard (*Chlamydotis undulata undulata*), and bittern (*Botaurus lentiginosus*) generate *boomings* that are very low frequency signals [43]. These low frequency signals fall near or below the threshold of human hearing (20 Hz). Birds have relatively greater hearing sensitivity than humans. For example, pigeons (*Columbidae*) have exceptional low-frequency (infrasound) perception [44]. However, current recording devices fail to fully capture these exceptional bird vocalisations. Accordingly, parallel to the development of birdsong recognisers there is a need of improving recorders and recording techniques.

In this study we have concentrated on birdsong, but automatic recorders also capture the sounds of other animals. In New Zealand for example, where introduced predators are responsible for the endangered status of many bird and reptile species, automatic recordings could be used to monitor population of these introduced animals. Our denoising technique would work well to prepare recordings for identification and estimation of abundance of species such as stoats (*Mustela nivalis*), feral cats (*Felis catus*), rats (*Rattus spp*) and dogs (*Canis familiaris*) whose calls are high frequency.

Song detection from long recordings and segmentation is another sub-topic in the field of birdsong recognition, especially when it comes to practical use. The segmentation method used to isolate the bird songs has a huge influence on both the recognition rate and recall rate of a recogniser. Conventional energy based segmentations done using the waveform would easily skip faded songs in the recordings mainly as a result of overlapping noise or the distance to the bird from the recording. On the other hand, this type of time domain approaches fail to separate bird songs from the background noise as they simply look at the energy and commonly a thresholding method to cutoff less energy sections. Therefore, this leads to increase false positives if the recogniser also fails to realise noise and discard them. But we speculate about using wavelet coefficients to do the segmentation in a more sophisticated manner. Separation of sound sources is another concept we did not consider in this context. Clearly it is not possible to separate sound sources easily in the presence of naturally recorded overlapping songs because the sounds are not linearly mixed even when we assume so, and the number of receivers (microphones) is always less than the number of sound sources. While the current study mainly focused on removing the stationary noise, it is essential to devise methods to tackle transient noise, but this would be more challenging because the birdsongs are also transient.

Future work is to be carried out extending the usability of wavelets to address aforementioned gaps in this research area. We are currently investigating different feature extraction methods including MFCC [45] and wavelet coefficients as well as potential machine learning algorithms and similarity measures for recognition and classification of birdsongs; it is important to determine the best combination of features that are strong enough to represent the birdsongs uniquely. The final goal is to develop a non-species specific, robust and user friendly automated platform for ecologists to automatically process natural field recordings collected using any recorder.

## Supporting Information

S1 Audio Bird call examples in Fig 8. (ZIP)

S2 Audio Birdsong examples in Fig 10. (ZIP)

S3 Audio Overlapped birdsong examples in Fig 12. (ZIP)

S4 Audio North Island robin song example in Fig 13. (ZIP)
